# Phantom validation of quantitative Y-90 PET/CT-based dosimetry in liver radioembolization

**DOI:** 10.1186/s13550-017-0341-9

**Published:** 2017-11-28

**Authors:** Marco D’Arienzo, Maria Pimpinella, Marco Capogni, Vanessa De Coste, Luca Filippi, Emiliano Spezi, Nick Patterson, Francesca Mariotti, Paolo Ferrari, Paola Chiaramida, Michael Tapner, Alexander Fischer, Timo Paulus, Roberto Pani, Giuseppe Iaccarino, Marco D’Andrea, Lidia Strigari, Oreste Bagni

**Affiliations:** 1 ENEA, Italian National Institute of Ionizing Radiation Metrology, Via Anguillarese 301, 00123 Rome, Italy; 2 Department of Anatomical, Histological, Forensic Medicine and Orthopedic Sciences, Sapienza University, Rome, Italy; 3 Nuclear Medicine Department, Santa Maria Goretti Hospital, Latina, Italy; 4 School of Engineering, Cardiff University, Cardiff, CF24 3AA United Kingdom; 5 Department of Medical Physics, Velindre Cancer Centre, Cardiff, UK; 6 ENEA, Radiation Protection Institute, Bologna Via Martiri di Monte Sole 4, 40129 Bologna, Italy; 7 GE Healthcare Medical Systems, Milan, Italy; 8 Sirtex, North Sydney, Sidney, NSW 2060 Australia; 9 Philips Technologie GmbH Innovative Technologies, Research Laboratories Pauwelsstr, 17, 52074 Aachen, Germany; 10 Depertment of Medico-surgical Sciences and Biotecnologies, Sapienza University of Rome, Rome, Italy; 11 Laboratory of Medical Physics and Expert Systems, Regina Elena National Cancer Institute, Via Elio Chianesi 53, 00144 Rome, Italy

**Keywords:** ^90^Y–PET, Liver radioembolization, Molecular radiotherapy, Dosimetry, Quantitative imaging

## Abstract

**Background:**

PET/CT has recently been shown to be a viable alternative to traditional post-infusion imaging methods providing good quality images of ^90^Y-laden microspheres after selective internal radiation therapy (SIRT). In the present paper, first we assessed the quantitative accuracy of ^90^Y-PET using an anthropomorphic phantom provided with lungs, liver, spine, and a cylindrical homemade lesion located into the hepatic compartment. Then, we explored the accuracy of different computational approaches on dose calculation, including (I) direct Monte Carlo radiation transport using Raydose, (II) Kernel convolution using Philips Stratos, (III) local deposition algorithm, (IV) Monte Carlo technique (MCNP) considering a uniform activity distribution, and (V) MIRD (Medical Internal Radiation Dose) analytical approach. Finally, calculated absorbed doses were compared with those obtained performing measurements with LiF:Mg,Cu,P TLD chips in a liquid environment.

**Results:**

Our results indicate that despite ^90^Y-PET being likely to provide high-resolution images, the ^90^Y low branch ratio, along with other image-degrading factors, may produce non-uniform activity maps, even in the presence of uniform activity. A systematic underestimation of the recovered activity, both for the tumor insert and for the liver background, was found. This is particularly true if no partial volume correction is applied through recovery coefficients. All dose algorithms performed well, the worst case scenario providing an agreement between absorbed dose evaluations within 20%. Average absorbed doses determined with the local deposition method are in excellent agreement with those obtained using the MIRD and the kernel-convolution dose calculation approach.

Finally, absorbed dose assessed with MC codes are in good agreement with those obtained using TLD in liquid solution, thus confirming the soundness of both calculation approaches. This is especially true for Raydose, which provided an absorbed dose value within 3% of the measured dose, well within the stated uncertainties.

**Conclusions:**

Patient-specific dosimetry is possible even in a scenario with low true coincidences and high random fraction, as in ^90^Y–PET imaging, granted that accurate absolute PET calibration is performed and acquisition times are sufficiently long. Despite Monte Carlo calculations seeming to outperform all dose estimation algorithms, our data provide a strong argument for encouraging the use of the local deposition algorithm for routine ^90^Y dosimetry based on PET/CT imaging, due to its simplicity of implementation.

## Background

Radioembolization with ^90^Y, or selective internal radiation therapy (SIRT), is a catheter-based therapy with the potential of delivering a high-radiation dose directly to liver tumors or metastases meanwhile minimizing the effects on healthy liver parenchyma [1–6]. The radiopharmaceutical consists of non-biodegradable ^90^Y-imprinted resin or glass-based microspheres with a diameter of approximately 20–40 μm. The tumoricidal effect is produced by beta particles emitted from ^90^Y incorporated on the surface of the resin matrix or integrally bound within the microsphere matrix, in the case of glass beads. The selectivity of SIRT is based on anatomic and physiological aspects of liver tumors and metastases that can be exploited for the selective delivery of the microspheres [7–9]. Microspheres are injected into the hepatic artery and delivered directly into the smaller blood vessels that feed the tumor, therefore being trapped within the tumor microvasculature.

It is worth noticing that in recent years, radioactive ^166^Ho poly(l-lactic acid) microspheres (166Ho-PLLA-MS) have been developed as a viable alternative to liver radioembolization with ^90^Y microspheres. In addition to high-energy beta-radiation, ^166^Ho emits gamma radiation that allows gamma camera quantitative imaging and dosimetry [10, 11]. Furthermore, holmium is highly paramagnetic and can be visualized using traditional magnetic resonance imaging (MRI), thereby allowing for MRI-based absorbed dose calculations [11–13].

^90^Y is a pure β-emitting radionuclide with maximum and average energies of 2.28 MeV and 933.7 keV, respectively. The corresponding maximum and average path lengths of the emitted β particles in soft tissue (1 g/cm^3^) are 11 and 2.5 mm. Although ^90^Y has been traditionally considered as a pure β^−^ emitter, the decay of this radionuclide has a minor branch to the 0^+^ first excited state of ^90^Zr at 1.76 MeV that is followed by a β^+^/β^−^ emission. This internal pair production has been largely studied in the past because it is generated by a rare electric monopole transition (E0) between the states 0^+^/0^+^ of ^90^Zr. A thorough explanation of the emission of β^+^ particles via internal pair production in the 0^+^–0^+^ transition of ^90^Zr is provided elsewhere [14].

In the last years, the small positronic emission has been exploited for ^90^Y–PET imaging studies after liver radioembolization, with the aim to provide quantitative post-treatment imaging and to prospectively improve the therapy [15–27]. Furthermore, the possibility of detecting β^+^ emissions from ^90^Y by PET scanners is paving the way for an accurate patient-specific dosimetry. Because microspheres can be assumed to be a permanent implant, the use of appropriate dose calculation algorithms allows quantitative ^90^Y–PET images to be converted into absorbed dose maps. The accuracy of dose calculation is of paramount importance in liver radioembolization as side effects and treatment outcome are related to absorbed doses. Over the last few years, there has been an intensifying debate revolving around the most accurate dosimetric approach for the assessment of the absorbed dose in molecular radiotherapy. Many authors obtained absorbed dose maps from ^90^Y–PET data performing three-dimensional convolution of the kernel function with the cumulated activity. A recent analysis by Pasciak et al. [26] proposed the use of local deposition technique, while previous studies used the analytical MIRD (Medical Internal Radiation Dose) approach.

The aim of this study is to investigate the impact of different computational approaches on absorbed dose calculation, starting from the quantitative accuracy of ^90^Y–PET. The workflow followed in the present work was structured into three logically sequential steps, which are detailed below.

(1) Firstly, the quantitative accuracy of reconstructed ^90^Y–PET data was evaluated using a cylindrical phantom and an IEC (International Electrotechnical Commission) body phantom for the assessment of recovery coefficients. Then, the PET camera’s ability to produce ^90^Y quantitative data was validated using an anthropomorphic phantom with a liver cavity and a background compartment. A hollow cylindrical insert was fixed into the liver cavity of the anthropomorphic phantom with the intent to simulate a hepatic lesion. Acquisitions were performed using a General Electric (GE) Discovery ST PET/CT scanner with a 6:1 tumor to background activity concentration ratio. The anthropomorphic phantom was acquired at days 1, 4, 5, 6, and 12 post-phantom preparation down to an activity concentration of 0.31 ± 0.02 MBq mL^−1^ for the lesion insert (initial activity concentration 5.5 ± 0.3 MBq mL^−1^) and 50 ± 3 kBq mL^−1^ for the liver background (initial activity concentration 0.89 ± 0.04 MBq mL^−1^). In the present phantom study, activity measurements were performed using a well-type radionuclide calibrator available on site and traceable to the Italian National Institute of Ionizing Radiation Metrology for the geometry being measured (accuracy within ± 5% at *k* = 2 level, as recommended by AAPM report 181 [28]). PET images were reconstructed using the standard GE algorithm (full 3D-OSEM VUEPoint HD two iterations, 15 subsets) and corrected for attenuation, scatter, random, and dead time using manufacturer software. Random and scatter corrections are incorporated into the iterative algorithm, with scatter correction performed using a fully 3D approach that considers both the axial and trans-axial scatter components.

(2) The second part of the study was devoted to absorbed dose calculations. Absorbed doses to the cylindrical insert were calculated from ^90^Y–PET images obtained in the previous stage, benchmarking different computational algorithms including (I) full Monte Carlo (MC) using Raydose [29]; (II) Kernel convolution using Philips Stratos [30]; (III) local deposition [26]; (IV) MC N-Particle code [31] (MCNP4c); and (V) MIRD analytical approach [32].

(3) Ultimately, absorbed doses calculated using MC codes were compared with those obtained performing experimental measurements with high-sensitivity LiF:Mg,Cu,P thermoluminescent dosimeters (TLDs) inside the same cylindrical insert filled with a homogenous ^90^YCl_3_ solution. LiF:Mg,Cu,P chips were fully characterized in terms of absorbed dose to water at the Italian National Institute of Ionizing Radiation Metrology (ENEA-INMRI) using the available ^60^Co reference gamma beam [33]. In the present study, the average absorbed dose to water obtained from TLD measurements was considered the gold standard.

The observation reported here provides key insight into dosimetry calculations related with ^90^Y–PET data. To the best of the authors’ knowledge, there are no published literature studies aimed to compare measured absorbed dose with dose values obtained from ^90^Y–PET quantitative data.

The results reported in the present paper are organized into three separate sections, reflecting the abovementioned workflow.

## Results

### Quantitative analysis

A cylindrical phantom, an IEC body phantom, and an anthropomorphic phantom were used to assess and validate the PET camera’s ability to image ^90^Y and produce quantitative data. ^90^Y-chloride in an aqueous solution of 0.1 mol dm^−3^ hydrochloric acid was used for phantom experiments as microspheres tend to settle over time during imaging, resulting in a non-uniform distribution of ^90^Y.

Adsorption of ^90^YCl_3_ on the inner walls of plastic phantoms may negatively affect PET quantitative imaging studies [34]. Therefore, in the present study, phantom preparation was performed using ultrapure water (18 MΩ cm) and adding diethylenetriaminepentaacetic acid (DTPA) at a concentration of 100 μg g^−1^ to the stock solution to prevent binding of ^90^Y to phantom polymethylmethacrylate (PMMA) walls. Activity was dispensed into the phantoms by accurate volume measurements performed using a calibrated four significant digit balance. Double weighing of the volume to be dispensed was performed for each phantom.

**Fig. 1:**
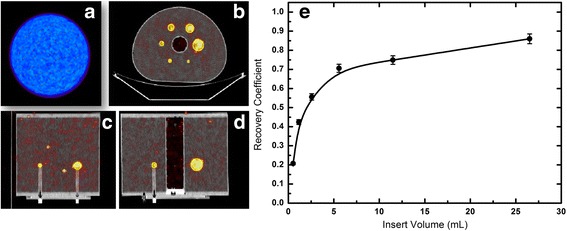
Quantitative accuracy of ^90^Y–PET acquisitions. **a** Cylindrical phantom used for validating PET calibration uniformly filled with an activity concentration of ^90^YCl_3_ at a concentration of 273 ± 14 kBq mL^−1^ at the time of acquisition. Diethylenetriaminepentaacetic acid (DTPA) at a concentration of 100 μg g^−1^ was used to prevent binding of ^90^Y to the phantom PMMA walls. An overnight (16 h) single bed acquisition was performed. **b** Transversal slice of the NEMA IEC/2001 phantom used for the assessment of recovery coefficients needed for partial volume corrections. RCs are defined as the ratio of the measured activity concentration to true activity concentration. The phantom is provided with six fillable spheres of varying volume (0.52, 1.15, 2.57, 5.57, 11.49, and 26.52 mL), each filled with an activity concentration of 2.28 ± 0.11 MBq mL^−1^ at the time of acquisition. Sagittal (**c**) and coronal (**d**) view of the same phantom are also shown. **e** Recovery coefficients as a function of the volume of the sphere. RCs lie in the range 0.2–0.86 for the smallest sphere and largest sphere, respectively. The relative uncertainty was estimated to be 5% of the RC value, corresponding to the relative uncertainty associated with the activity as measured by the clinical radionuclide calibrator

The ^90^Y calibration factor on our GE Discovery DST PET/CT scanner was evaluated rescaling the ^18^F calibration factor by the ^90^Y beta plus branch ratio (0.00318%). This procedure was validated performing an overnight acquisition of a cylindrical phantom (5640 mL volume, Fig. 1a) uniformly filled with (1.53 ± 0.08) GBq of ^90^YCl_3_ mixed with pure water (273 ± 14 kBq mL^−1^). The phantom was positioned at the center of the field of view (FOV) and a 16-h single bed acquisition was launched. After the acquisition, data were reconstructed using two iterations, 15 subsets. Past research on the same Discovery ST PET/CT scanner [16] showed that two iterations represent a good trade-off between signal recovery and image noise on object with volume larger than 10 mL, approximately. Images were elaborated with the built-in PET VCAR GE software. The recovered concentration in the uniform phantom was 257 ± 16 kBq mL^−1^ (6.2% standard deviation) with an underestimation of − 5.9% with respect to the true concentration (Table 1). This finding confirms the validity of the proposed calibration procedure as the true and the measured activity concentrations agree well within the stated uncertainties.

**Table 1:** Validation phantom. Recovered activity concentration in the uniform cylindrical phantom after an overnight acquisition

True activity concentration (MBq mL^−1^)	Recovered activity concentration in the uniform phantom (MBq mL^−1^)	Scan time (min)	Reconstruction parameters	Homogeneity index
0.273 ± 0.008	0.257 ± 0.016	960	2i, 15s	0.07

Partial volume effect (PVE) was studied through the assessment of absolute recovery coefficients (RCs) on spherical objects, i.e., the ratio of the measured activity concentration to true activity concentration. Regions of interest (ROI) as large as the physical diameter of the sphere were drawn. A NEMA IEC/2001 phantom with six fillable spheres of varying volume (0.52, 1.15, 2.57, 5.57, 11.49, and 26.52 mL) was used for the assessment of RCs (Fig. 1b–d). Each sphere was filled with a uniform ^90^YCl_3_ liquid solution having an activity concentration of 2.28 ± 0.11 MBq mL^−1^ at the time of acquisition. An 8:1 sphere-to-background ratio was used, as recommended by the NEMA Standards Publication NU 2-2007 [35]. PVE led to significant underestimation of the activity concentration, especially in spheres with a volume below 10 mL, approximately. RCs range from 0.2 for the smallest sphere to 0.86, approximately, for the largest sphere (Fig. 1e).

**Fig. 2:**
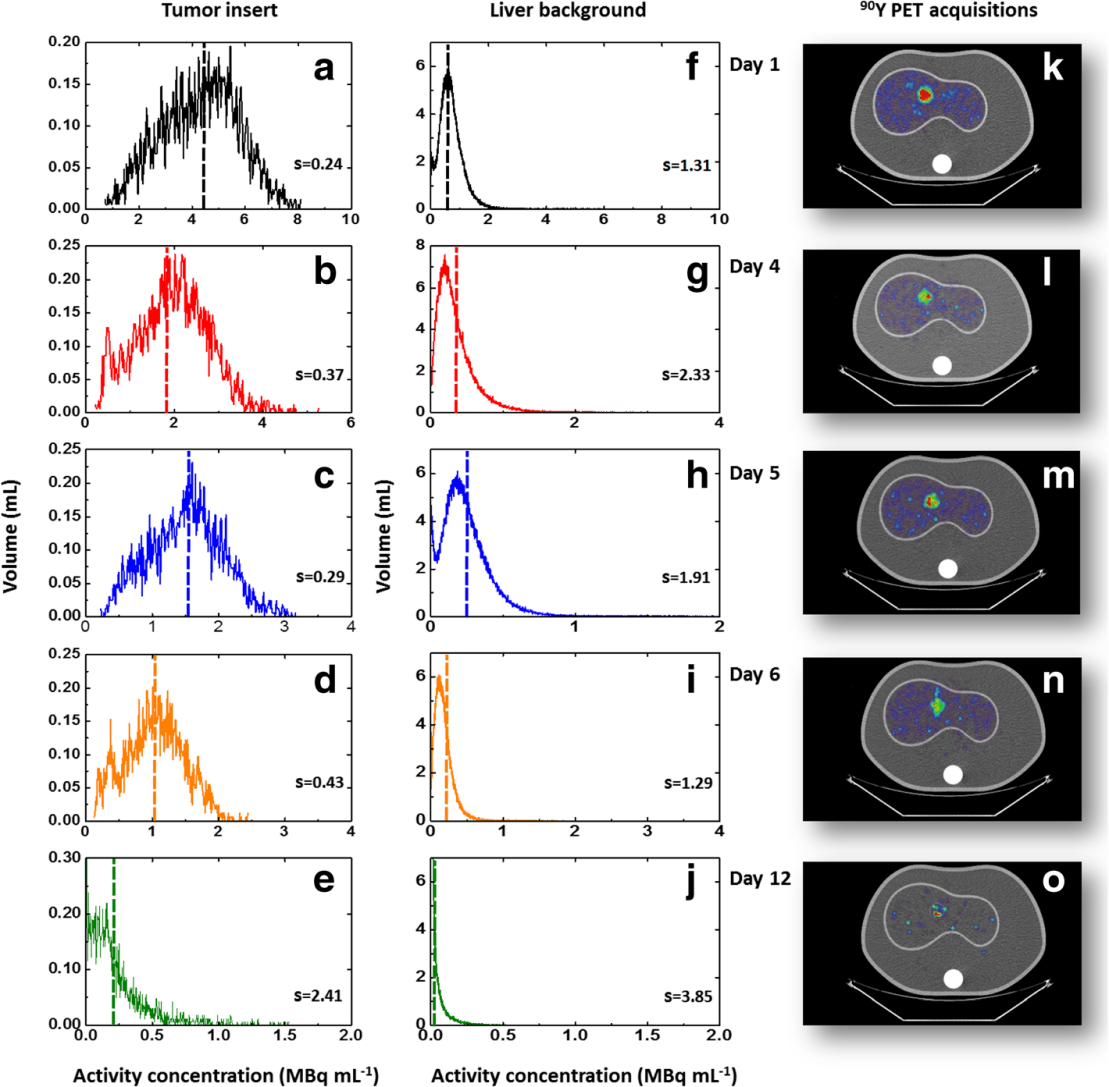
Differential activity concentration volume histograms (dAVH). ^90^Y–PET images were reconstructed using a GE Discovery ST PET/CT system with 2.73 × 2.73 × 3.27mm^3^ detector elements. A full 3D-OSEM VUEPoint HD reconstruction algorithm, two iterations, 15 substeps, was used. Acquisitions at day 1 and 4 were performed using 15 min scan time, while 30 min acquisition time was used for imaging at days 5, 6, and 12. Acquisition duration was varied over time as we aimed to perform acquisition in realistic clinical conditions. Scan time below 30 min are very unlikely in the clinical practice for activity concentrations below 2 MBq/mL, especially in the absence of a TOF scanner. Activity distributions of ^90^Y–PET images were analyzed in terms of dAVHs. Positive skewness (indicated with s) was obtained for all dAVHs. **a**–**e** dAVH of the cylindrical insert fixed into the liver cavity of the anthropomorphic phantom. Heterogeneous activity distributions were obtained for all acquisitions. **f**–**j** dAVH of the liver background. Visual inspection of the images (**k**–**o**) showed the presence of hot and cold spots in the insert, supposed to be uniformly filled with ^90^Y. As a general rule, both for the tumor insert and the liver compartment the lower the activity concentration, the higher the skewness value

**Fig. 3:**
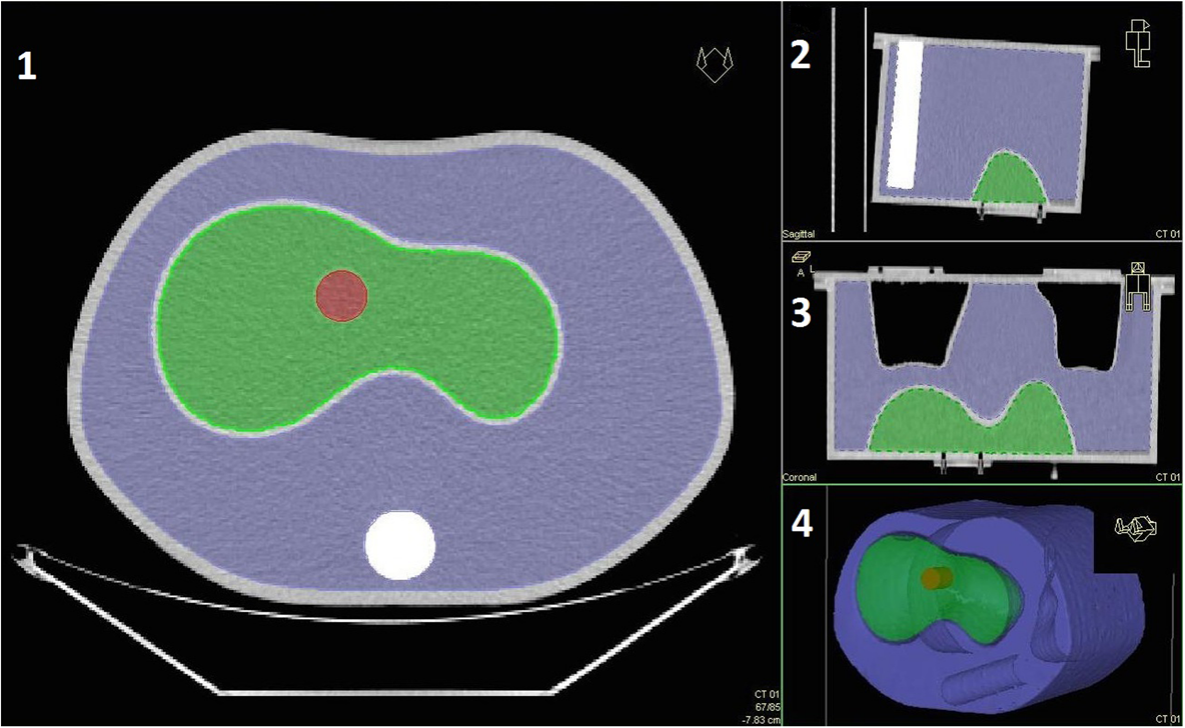
Anthropomorphic phantom used to validate the PET camera’s ability to produce ^90^Y quantitative data. The phantom is composed by a background region (10,300 mL), a liver compartment (1200 mL), left (900 mL) and right lung (1100 mL), and a spine insert (200 mL). A homemade cylindrical PMMA insert was manufactured and fixed into the liver cavity to simulate a hepatic lesion. The nominal volume of the insert is 19.13 mL (diameter 28.5 mm, height 30 mm)

The PET camera’s ability to produce ^90^Y quantitative data was validated using an anthropomorphic phantom with a liver cavity and a background compartment. A homemade cylindrical PMMA insert was manufactured and fixed into the liver cavity to simulate a hepatic lesion. The nominal volume of the insert is 19.13 mL (diameter 28.5 mm, height 30 mm). Measurements in anthropomorphic geometry were performed using a 6:1 tumor to background activity concentration ratio, as this uptake ratio is often encountered in the clinical practice. The first scan was performed with an activity concentration of 5.5 ± 0.3 MBq mL^−1^ for the lesion insert and 0.89 ± 0.04 MBq mL^−1^ for the liver. The anthropomorphic phantom was then acquired at days 4, 5, 6, and 12 (according to the scanner availability) down to an activity concentration of 0.31 ± 0.02 MBq mL^−1^ for the lesion insert and 50 ± 3 kBq mL^−1^ for the liver. The background compartment of the phantom was filled with non-radioactive water. Activity distributions of ^90^Y–PET images were analyzed in terms of differential activity concentration volume histograms (dAVHs), generated by binning the activity concentration values from each voxel in the volume and calculating the volume of structure containing an activity concentration given by bin:

1 $$ dAVH=-\frac{\Delta}{\Delta \mathrm{c}}\left(\frac{v_i\left({c}_i\right)}{V}\right)=-\frac{\mathrm{d}}{\mathrm{d}\mathrm{c}}\left(\frac{v(c)}{V}\right) $$

where Δ*c* is the binning resolution in terms of activity concentration, *v*_*i*_ is the *i-*th volume element containing an activity concentration of at least *c*_*i*_, and *V* is the total volume. dAVHs of the tumor insert are reported in Fig. 2, while Fig. 3 shows the anthropomorphic phantom used to perform ^90^Y–PET quantitative acquisitions. Despite the cylindrical insert being uniformly filled with ^90^YCl_3_, heterogeneous activity distributions were obtained from ^90^Y–PET data. As a general rule, visual inspection of the images showed the presence of hot and cold spots in regions of the phantom uniformly filled with ^90^Y (Fig. 2k–o). From a quantitative point of view, positive-skewed dAVHs were obtained for all acquisitions, i.e., activity concentration values clustered more toward lower activity concentration values with few higher values (Fig. 2a–j). This is in keeping with previous research on noise in PET imaging, modeling positive-skewed noise probability density function with Poisson, negative binomial, log-normal, and gamma distributions [36]. Consistently, we found that the lower the number of detected β^+^ events (i.e., the noise level), the higher the skewness value (Fig. 2a–j). A comprehensive study of noise in ^90^Y-PET imaging lies outside the scope of the present study. The reader is referred to Carlier et al. [37] for details.

**Fig. 4:**
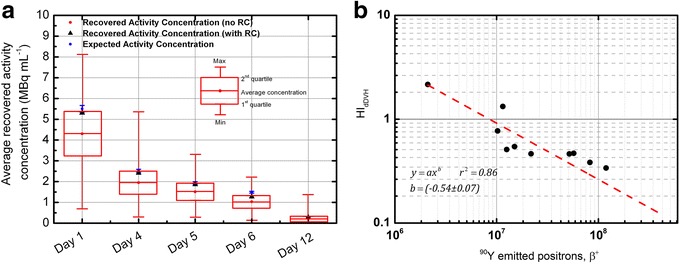
Quantitative analysis. **a** Recovered activity concentration in the tumor insert of the anthropomorphic phantom. Data are presented in terms of minimum and maximum recovered activity values, 1st and 3rd quartile, average activity concentration and recovered activity concentration after correcting for PVE. After the application of RCs, differences with measured activity concentration values reduce significantly with underestimations of the order of 4% down to an activity concentration of about 2 MBq mL^−1^. **b** For each phantom acquisition HI_dDVH_ were plotted as a function of the number of positrons emitted from the VOI. A power function of the form *y* = *ax* ^*b*^ was used to fit data points, where *x* is the number of emitted positrons from the VOI and *a* and *b* coefficients to be determined from the fit. From the regression analysis it resulted *b* = − 0.54 ± 0.07 (with *r* ^2^ = 0.86)

A systematic underestimation of the recovered activity, both for the tumor insert and for the liver background, was found. This is particularly true if no partial volume correction is applied through RCs. After the application of RCs, differences with measured activity concentration values reduce significantly with underestimations of the order of 4% down to an activity concentration of about 2 MBq mL^−1^ (Table 2 and Fig. 4a). For lower activity concentration values, deviations in the range 10–15% were obtained down to 0.24 MBq mL^−1^ (Tables 2 and 3). PVE typically occurs whenever the object size is less than three times the full width at half maximum of the reconstructed image resolution [38]. Therefore, PVE in the liver were assumed negligible.

**Table 2:** Recovered activity in the tumor insert before and after compensation for partial volume effect through recovery coefficients. Recovered activity concentrations are compared to measured values

Day of scan	True activity concentration (MBq mL^−1^)	Average recovered concentration (MBq mL^−1^) [1st–3rd quartile] (no recovery coefficient applied)	Average recovered concentration (MBq mL^−1^) [% deviation] (recovery coefficient applied)	Homogeneity Index	Reconstruction parameters	Scan Time (min)
1	5.50 ± 0.16	4.30 [3.24–5.36]	5.3 [− 3.6%]	0.34	2i, 15s	15
4	2.52 ± 0.08	1.95 [1.4–2.51]	2.41 [− 4.4%]	0.42	2i, 15s	15
5	1.94 ± 0.06	1.51 [1.12–1.88]	1.86 [− 4.1%]	0.36	2i, 15s	30
6	1.49 ± 0.04	1.03 [0.72–1.34]	1.27 [− 14.7%]	0.42	2i, 15s	30
12	0.31 ± 0.01	0.22 [0.076–0.294]	0.27 [− 12.4%]	1.01	2i, 15s	30

**Table 3:** Recovered activity in the liver background. Recovered activity concentrations are compared to measured values

Liver Background	True activity concentration (MBq mL^−1^)	Recovered concentration (MBq mL^−1^) Average, [1st–3rd quartile] [% deviation]	Homogeneity Index	Reconstruction parameters	Scan Time (min)
1	0.89 ± 0.03	0.76 [0.430–0.920] [− 14.6%]	0.84	2i, 15s	15
4	0.41 ± 0.01	0.37 [0.160–0.460][− 9.8%]	0.94	2i, 15s	15
5	0.31 ± 0.01	0.26 [0.140–0.330] [− 16.1%]	0.91	2i, 15s	30
6	0.24 ± 0.01	0.19 [0.090–0.240] [− 20.8%]	0.90	2i, 15s	30
12	0.051 ± 0.002	0.021 [0.004–0.030][− 58.8%]	2.80	2i, 15s	30

A homogeneity index, *HI*_*dAVH*_, was derived from dAVHs to quantify the dispersion of the average activity within the volume of interest (VOI) [39]:

2 $$ {HI}_{dAVH}=\frac{1}{C_{mean}}\sqrt{\sum \frac{v_i}{V}\cdot {\left({c}_i-{C}_{mean}\right)}^2} $$

where *C*_*mean*_ is the average activity concentration over the selected VOI and *c*_*i*_ the activity concentration at the voxel level. For each acquisition, *HI*_*dAVH*_ were plotted as a function of the number of positrons emitted from the VOI (Fig. 4b). Calculated *HI*_*dAVH*_ are reported in Tables 1, 2, and 3. A power function of the form *y = ax* ^*b*^was used to fit data points, where *x* is the number of emitted positrons from the VOI and *a* and *b* coefficients to be determined from the fit. From the regression analysis, it resulted *b = −* 0.54 ± 0.07 (with *r* ^2^ *=* 0.86), indicating that the homogeneity index is representative of image noise (thereby associated with low counting statistics) and it is likely to decrease in proportion to the square root of the number of emitted positrons.

### Absorbed dose calculations

The absorbed dose to the tumor cylindrical insert (Fig. 2k–o) was calculated using different computational approaches, namely: I) Full MC using Raydose II) Kernel-convolution using Philips Stratos III) Local deposition (LD) algorithm IV) MC N-Particle code (MCNP4c), assuming uniform activity distribution V) MIRD analytical approach. Methods I–III use ^90^Y-PET images generated from the PET scanner for the assessment of the absorbed dose at the voxel level. Therefore differential dose volume histograms (dDVH) of the VOI can be produced and compared. Methods IV–V allow the average absorbed dose to the tumor insert to be determined assuming that the cylindrical insert is uniformly filled with a known ^90^Y activity concentration. Therefore, possible activity concentration non-uniformities are not considered.

**Fig. 5:**
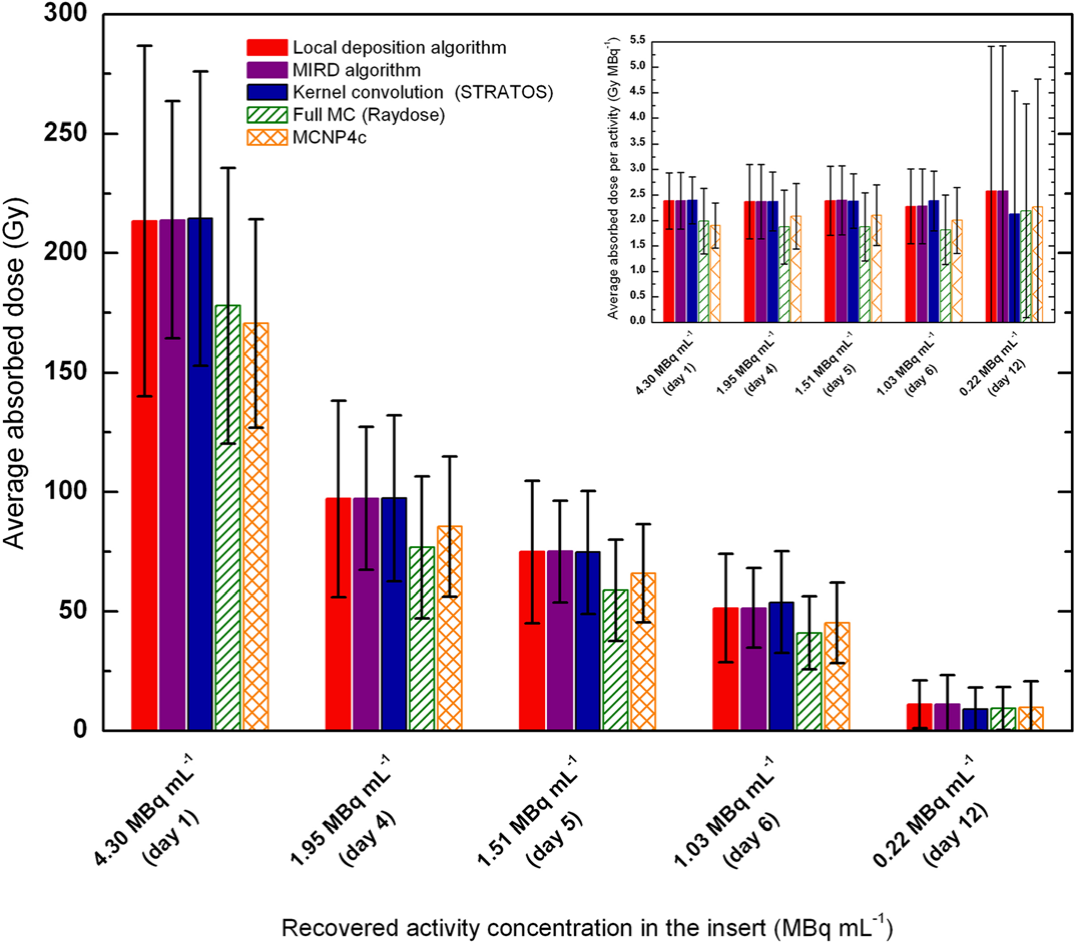
Comparison of absorbed doses. Different dose calculation algorithms were used to compare average absorbed doses, namely (I) LD algorithm (red), (II) MIRD analytical approach (violet), (II) Kernel convolution using Philips Stratos (blue), (IV) full MC using Raydose (green stripe pattern), (V) MCNP4c (orange crosshatch pattern). The vertical bar chart displays the mean absorbed dose and its standard deviation. For methods I-II-II, the standard deviation was assessed from dose-volume-histograms obtained after calculation. For methods IV and V, the activity of ^90^Y contained in the cylindrical insert was recovered from ^90^Y–PET images using the dAVH approach described by Eq. 1. Then, the mean activity value, along with its standard deviation, has been used to assess doses with methods IV and V, thus providing a mean dose ± standard deviation. MC-based dose evaluations are reported with a different pattern to differentiate MC calculations with other algorithms that do not directly account for the presence of the acrylic wall. Average absorbed doses are reported along with their standard deviations. All dose algorithms provided comparable dose estimates with deviations below 9%, except for dose calculations performed on day 1, where the standard deviation over the average absorbed dose values is 20%. Insert: comparison between average absorbed dose per unit activity into the cylindrical insert. When the activity concentration decreases down to 0.22 MBq/mL (day 12) the standard deviation is approximately 100% the dose value

From a clinical point of view, microspheres become a permanent implant. Therefore, dose calculations were performed considering a single image (or activity concentration value) and assuming that the entire dose is delivered over the ^90^Y physical half-life of 2.67 days. A comparison of the calculated average absorbed dose is shown in Fig. 5, where the different dose calculation algorithms were applied to each of the clinical condition reported in Fig. 2k–o. It is worth noticing that the possible influence of the wall thickness is considered only in Raydose and MCNP simulations. Raydose uses CT units to determine attenuation coefficients for radiation transport; therefore, perturbation effects of local heterogeneities (e.g., PMMA phantom walls) are correctly accounted for. Equivalently, a cylinder provided with a 1-mm thick PMMA lateral wall (*ρ* = 1.19 g cm^−3^) was implemented in MCNP simulations (elemental composition by weight: H = 0.080538, C = 0.599848, and O = 0.319614). Therefore, MC-based dose evaluations are reported with a different pattern to differentiate MC calculations with other algorithms that do not directly accounts for the presence of the acrylic wall.

As a general rule, all dose algorithms provided comparable dose estimates with deviations within 9%, except for dose calculations performed on day 1, where the standard deviation over the average absorbed dose values is 20%. When compared to each other, LD, MIRD approach, and Kernel-convolution methods provided comparable absorbed doses with an average deviation within 6% (maximum deviation 12% on day 1). Similarly, MC codes provided comparable absorbed dose values to the tumor insert, with an average deviation below 4%. Calculations performed using kernel convolution algorithm provided dose estimates in excellent agreement with the well-known MIRD calculation method, with differences below 5% for all acquisitions, except for day 12 where the difference in the average absorbed dose is 17.7%.

**Fig. 6:**
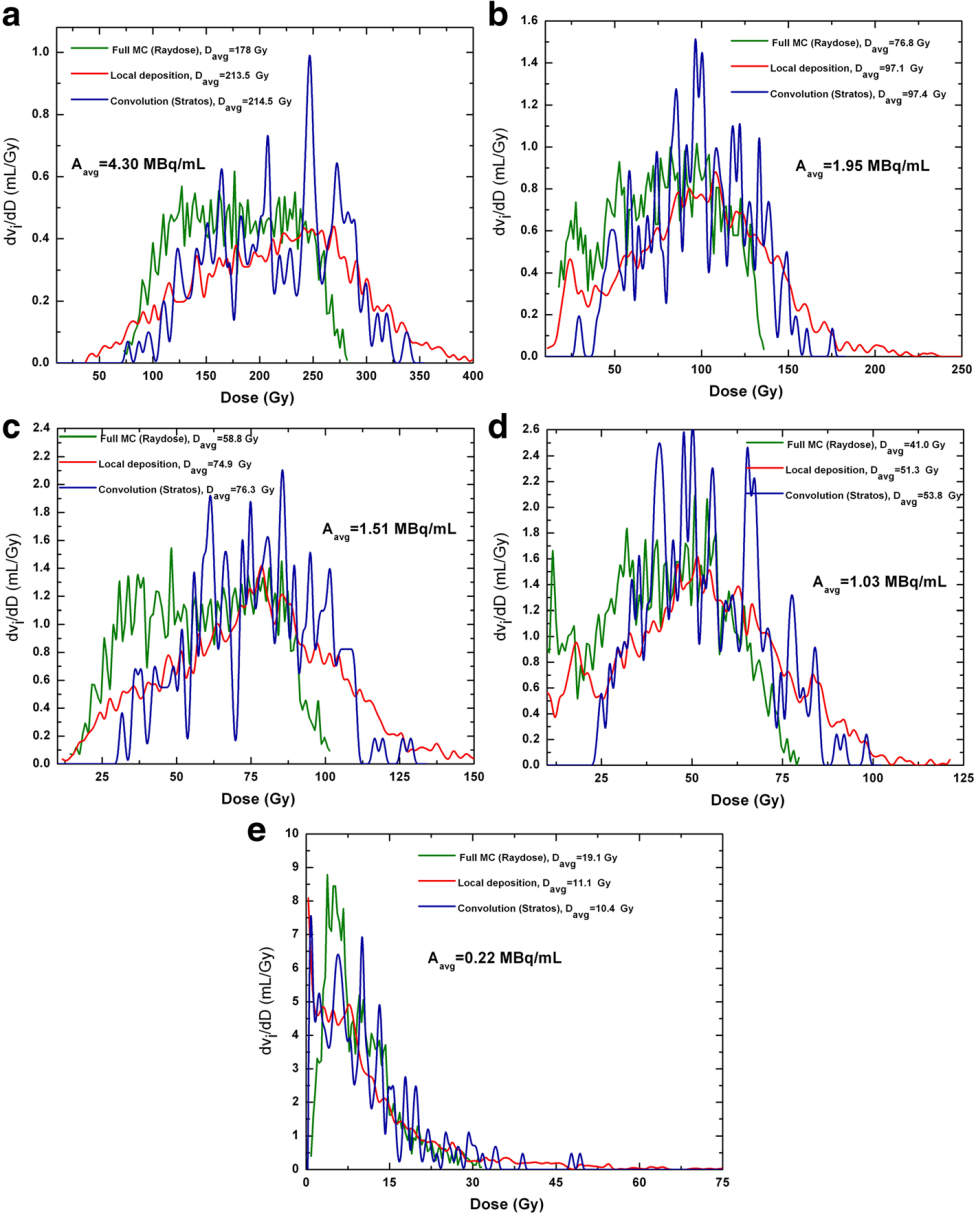
**a**–**e** Comparison of differential DVHs obtained in the cylindrical insert using the three voxel-based dose algorithms. (I) Full Monte Carlo calculations with Raydose, (II) LD algorithm, and (III) convolution approach using Philips Stratos. Results are presented in terms of dv_i_/dD, i.e., absolute volume element (dv_i_) receiving dose in the corresponding dose bin (dD). The integral under each differential DVH yields the insert volume (nominal volume 19.13 mL). VOI contouring performed using methods I and II provided a volume of 19.60 mL (+ 2.45%), while insert contouring performed with Philips Stratos resulted in a volume of 19.21 mL (+0.42%). The quantity *A*_avg_ represents the average insert activity concentration assessed from image-based evaluations. Real activity concentrations, measured using the available dose calibrator, are reported in Table 2

Owing to the non-uniformity in the activity distribution, the standard deviation of each dose point is generally large (of the order of 30–40% of the dose value) and becomes unacceptable when the activity concentration decreases down to 0.22 MBq/mL (approximately 100% the dose value). Figure 6 shows a comparison between dDVHs generated by full MC calculations with Raydose, LD algorithm, and convolution approach with Philips Stratos. dDVHs were produced after a voxel-based dose estimation of the cylindrical insert. In the present study, the DICOM Structure Set format (RTSTRUCT) module was used to transfer phantom structures and related data between the workstations. Insert segmentation was performed so as to exclude the PMMA wall from the contour. Results in Fig. 6 are presented in terms of dv_i_/dD, i.e., absolute volume element (dv_i_) receiving dose in the corresponding dose bin (dD). The integral under each differential DVH yields the insert volume (nominal volume 19.13 mL). VOI contouring performed using methods I and II provided a volume of 19.60 mL (+ 2.45%), while insert contouring performed with Philips Stratos resulted in a volume of 19.21 mL (+ 0.42%).

### Absorbed dose measurement using LiF:Mg,Cu,P TLDs

**Fig. 7:**
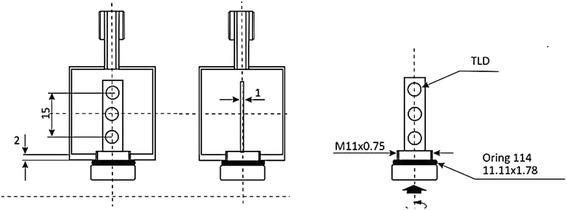
Technical drawing of the cylindrical phantom containing TLD chips surrounded by ^90^YCl_3_ aqueous solution. Custom PMMA cylindrical inserts were manufactured at ENEA-INMRI to perform absorbed dose measurements with LiF:Mg,Cu,P thermoluminescence detectors

Absorbed dose calculations presented in the previous section were validated by absorbed dose to water measurements using LiF:Mg,Cu,P chips inside a PMMA phantom filled with a homogenous ^90^YCl_3_ aqueous solution. Therefore, the average absorbed dose to water obtained from TLD measurements was considered the gold standard against which other dose algorithms can be benchmarked. Each TLD had 4.5 mm diameter and was 0.8 mm height. To this purpose, measurements were performed manufacturing six cylindrical phantoms the size of that used for quantitative analysis (Fig. 7). Each cylindrical phantom hosted a waterproof PMMA stick containing 3 TLD chips encapsulated by a polystyrene envelope (for a total of 18 chips). The PMMA phantoms were manufactured so that the radioactive liquid environment surrounds the whole stick. The ^60^Co reference gamma beam available at ENEA-INMRI was used to evaluate the calibration coefficient of each TLD chip in terms of absorbed dose to water. Relative standard uncertainty on the TLD calibration coefficient was found to be in the range 0.7–2.2%.

The measurement of the absorbed dose from beta-emitting radionuclides in liquid solution is not straightforward. The exclusion of radioactivity from the volume occupied by the TLD is a major effect that needs to be accounted for. Furthermore, the waterproof polystyrene envelope unavoidably contributes to beta particle attenuation during the measurement. Finally, as TLD calibration was performed using a source and a geometry different from the real experimental condition, an energy and geometry correction factor needs to be considered. An accurate description of the measurements equation is reported in the “Methods” section, along with a detailed description of correction and conversion factors.

PMMA samples were filled with ^90^YCl_3_ aqueous solution with activity concentration of 6.7 MBq/mL, approximately. Activity concentration was determined by measuring an aliquot of the stock solution in terms of activity per unit mass. Then, this was used to determine the activity of all subsequent sources produced from this stock solution. Accurate measurements of ^90^Y activity concentration were performed using the ENEA-INMRI portable triple-to-double coincidence ratio (TDCR) counter [40], with a relative standard uncertainty below 1%. Measurement time was 30 min approximately for each sample.

After measurement, each stick was removed from the sample and TLD chips were manually removed from the PMMA holder using dedicated tongs. One out of six sticks holding the chips was wasted because of leaks in the PMMA envelope that caused radioactive water to get in contact with the chips. Furthermore, 8 TLD chips were accidentally slightly contaminated during the removal procedures. These data points were removed from the final data as significantly higher.

The average absorbed dose to water per emitted beta particle was finally assessed as the weighted average of TLD measurements and resulted to be (1.09 ± 0.07) × 10^−11^ Gy/particle, with an associated relative weighted standard uncertainty of 6.3%. The measured value can be compared with the absorbed dose to water per emitted beta particle obtained using two MC codes, namely MCNP4c and Raydose.

With MCNP4c, the absorbed dose to water was calculated implementing the full cylindrical phantom geometry into the code. A homogenous ^90^Y source was considered into the phantom, and the delivered energy to water was scored into geometrical cells the size of a TLD chip. Simulations provided absorbed dose values per unit emitted beta particle of (1.010 ± 0.005) × 10^−11^ Gy/particle.

**Fig. 8:**
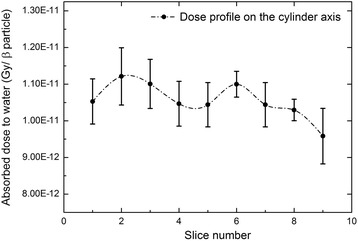
Profile of the absorbed dose to water per emitted beta particle on the cylinder axis, as calculated by Raydose. Voxel-by-voxel absorbed dose calculations were performed using the acquisition in anthropomorphic geometry reported in Fig. 2k. VOIs the size of a TLD (4.5 mm diameter, 0.8 mm height) were drawn on the dose profile. The average absorbed dose per unit emitted beta particle was determined to be (1.06 ± 0.04) × 10^−11^ Gy/particle

On the other hand, image-based calculations with Raydose were performed using the ^90^Y-PET acquisition reported in Fig. 2k. Voxel-by-voxel absorbed dose calculations were performed on the cylindrical insert. Finally, VOIs the size of a TLD chip (4.5 mm diameter, 0.8 mm height) were drawn on the dose profile (Fig. 8). The average absorbed dose per unit emitted beta particle was determined to be (1.06 ± 0.04) × 10^−11^ Gy/particle.

Overall, absorbed dose assessed with MC codes is in excellent agreement with those obtained using TLD in liquid solution, thus confirming the soundness of both calculation approaches (Table 4). This is especially true for Raydose, which provided an absorbed dose value within 3% of the measured dose, well within the stated uncertainties.

**Table 4:** Absorbed dose to water measured and calculated by MCNP4c and Raydose MC codes. ${\overline{\mathrm{D}}}_{\mathrm{w}}^{\mathrm{meas}}$ and ${\mathrm{D}}_{\mathrm{w}}^{\mathrm{MCNP}}$ are in the ratio 1.08, while ${\overline{\mathrm{D}}}_{\mathrm{w}}^{\mathrm{meas}}$ and ${\mathrm{D}}_{\mathrm{w}}^{\mathrm{Raydose}}$ are in the ratio 1.03, well within the stated uncertainties. ${\overline{\mathrm{D}}}_{\mathrm{w}}^{\mathrm{meas}}$: absorbed dose per emitted beta particle measured using LiF:Mg,Cu,P TLDs. ${\mathrm{D}}_{\mathrm{w}}^{\mathrm{MCNP}}$: absorbed dose per emitted beta particle calculated using MCNP4c in analytical geometry. ${D}_w^{Raydose}$: absorbed dose per emitted beta particle calculated running Raydose on ^90^Y–PET images

	Average absorbed dose to water	Relative weighted standard uncertainty (%)
Measurements, ${\overline{D}}_w^{meas}$	(1.09 ± 0.07) × 10^−11^ Gy/particle	6.3
MCNP-4c, ${D}_w^{MCNP}$	(1.010 ± 0.005) × 10^−11^ Gy/particle	0.5
Raydose, ${D}_w^{Raydose}$	(1.06 ± 0.04) × 10^−11^ Gy/particle	3.8

## Discussion

This study offers key insight into how different approaches to dosimetry calculations can accurately assess the absorbed dose after liver radioembolization using ^90^Y–PET data. At a time of increasing evidence for an absorbed radiation dose-effect relationships in radioembolization with ^90^Y-laden microspheres [41, 42], it is the authors’ belief that there is an urgent need for accurate dosimetry in patients undergoing SIRT therapy. In fact, it is generally accepted that the absorbed dose both to tumor areas and to healthy liver is likely to have a significant impact on treatment’s clinical effectiveness. Furthermore, since microspheres administered during therapy remain trapped within the microvasculature, imaging requirements reduce to a single-time acquisition, thus simplifying the whole dosimetry process. As a consequence, there is great potential for SIRT dosimetry to become routine in the clinical practice.

Patient-specific dosimetry in SIRT suffers from a number of image-degrading effects. Besides PVE, that is likely to play a major role in small lesions, low true coincidence statistic due to the combined interplay of low beta plus branching ratio and low achievable acquisition time are likely to impact the image quality. Furthermore, the high scatter component intrinsic of ^90^Y PET imaging may negatively affect image quantification. Ultimately, patient respiratory motion is the primary cause of image blurring possibly leading to systematic dose underestimations [43].

In this study, we concerned ourselves with quantitative accuracy of ^90^Y PET and the impact that different dose calculation approaches have on image-based dosimetry after liver radioembolization. Yet another question of interest for us was to compare absorbed doses obtained from MC calculations with those obtained through absorbed dose measurements performed with TLD chips into a liquid radioactive environment. To the best of our knowledge, this line of research is the first to investigate the degree of agreement between calculated and measured dose using ^90^Y–PET quantitative data.

Our data confirm and expand previous observations. Since most scanner do not support ^90^Y as a viable radionuclide option, scanner calibration should be performed using surrogate radionuclides and applying proper decay correction factors (e.g., ^86^Y [44, 45] or ^22^Na [46]). Quantitative analysis showed that partial volume effects dominate spheres of volume below 11.5 mL (diameter < 28 mm), approximately. Recovery of activity concentration measured in the largest sphere on day 0 of imaging underestimated the true activity concentration of − 15%, approximately. Our results are in keeping with previous studies [18, 47]. However, the recent QUEST study [48] suggests that ToF-PET scanners are likely to improve contrast of hot spheres and increase RCs.

Another important consideration is that the low branching ratio for ^90^Y positron emission entails long scan times in order to reduce image noise. This is especially true for non-ToF–PET scanner, where the acquisition time plays a key role. Quantitative analysis in anthropomorphic geometry showed that despite the tumor insert being uniformly filled with a homogenous ^90^YCl_3_ solution, ^90^Y–PET images presented significant spatial non-uniformity. Notwithstanding this, for activity concentrations exceeding 1.5 MBq/mL, the recovered activity concentration underestimated the known value by 4% approximately, demonstrating that accurate quantitation of ^90^Y is possible if long scan time is performed and if partial volume effects are accurately compensated for. Quantitative accuracy decreases for decreasing activity concentration levels. Interestingly, our results are in close agreement with those obtained by Mille et al. [49] which recovered 95.4% of the known activity in a large cylindrical phantom despite obtaining noisy images with a voxel variability of 21%.

In the present study, the degree of uniformity was quantified in terms of homogeneity index. Quantitative data demonstrated a noise-like behavior of the homogeneity index, with the latter decreasing in proportion to the square root of the number of emitted positrons. This finding suggests that for the considered geometry and settings, image non-uniformities are mainly imputable to the low-counting statistics. It is interesting to note that activity underestimation both in the tumor insert and in the liver compartment is associated with increasing homogeneity index.

Of additional concern has been the impact of different dose calculation algorithms in anthropomorphic geometry. As a matter of fact, to date only a few studies attempted to compare different dosimetry methods in liver radioembolization. Mikell and co-workers [50] compared four voxel-based dosimetry dose algorithms based on ^90^Y bremsstrahlung SPECT images (namely, MC, soft-tissue kernel with density correction (SKD), soft-tissue kernel (SK), and LD method). Interestingly, the authors found that for tumor, non-tumoral liver mean absorbed doses calculated with SKD, SK, and LD are equivalent to MC (within 5%). Deviations in the mean absorbed dose values increase when dosimetry is performed in the lungs, with right lung dosimetry being strongly influenced by the liver–lung interface [50]. In another work, Grassi et al. [51] compared STRATOS with a homemade software package (VoxelMed), both in phantom and in patients undergoing radiopeptide therapy with ^177^Lu. Both software performed well, with an agreement within 5% in phantom. Larger deviations were observed in patients.

In the present study, the large voxel variability of absorbed dose values is related to the abovementioned intrinsically noisy nature of ^90^Y PET images. However, on average, absorbed doses to the tumor insert have been found to be consistent to within 9%, except for dose calculations performed on day 1, where the standard deviation over the average absorbed dose values is 20%.

The most striking observation to emerge from the data comparison is the difference between dose values obtained with MC codes (namely, MCNP and Raydose) and other algorithms. The foremost cause of the slightly lower dose values obtained with Raydose is most probably due to the resampling of the CT grid from 512 × 512 to 128 × 128, executed for efficiency purposes. The larger voxel size, together with the inhomogeneity in the underlying PET images, produced a smoothing of the activity data. This is evident in the fine distribution of the dose (Fig. 6a–e): DVHs obtained with Raydose do not present any high-dose tail as any hot spots in a single voxel are shared with adjoining voxels. This is likely to produce lower doses especially at the liquid–PMMA interface. On the other hand, the lower doses obtained using MCNP could be interpreted as being a result of: (i) the input activity concentration, supposed to be uniformly distributed over the entire cylindrical object and (ii) the absence of spill-in activity from regions around the cylindrical insert in MCNP simulations. This is likely to result in a lower dose to the cylindrical insert.

Furthermore, when interpreting the results of the current study, it is also important to note that the presence of the PMMA wall of the cylindrical insert may had an impact on dose calculations, possibly leading to biased estimates between methods that directly account for the acrylic edges (Raydose, MCNP) and those that do not (kernel convolution, LD, and MIRD). The impact of the PMMA wall on dose calculations is somehow expected and inevitable, given the non-negligible presence of acrylic material (4.98 g) if compared to the total mass of the insert when filled with ^90^YCl_3_ (24.1 g). From a computational perspective, there is no substantial material difference between tumor and background liver. As a consequence, this effect is not expected in a clinical scenario, unless dose calculations are performed at the liver–lung interface, where difference in material density may play a key role [50].

Of note, average absorbed doses determined with the LD method are in excellent agreement with those obtained using the MIRD and the kernel convolution dose calculation approach. This result provides a strong argument for encouraging LD algorithm for the evaluation of absorbed doses in the clinical practice, where resource–intensive software packages are not always available. Similar conclusions have been drawn in recent research on the matter [26]. However, the large dose variability at the voxel level—most likely due to a scenario with low true coincidences and high random fraction—raises questions about the possibility of using dDVH for radiobiological modeling if acquisitions are performed with a non-TOF scanner.

During the last 20 years, there has been increasing interest in the measurement of absorbed doses from internal beta emitters used in molecular radiotherapy. In particular, TLDs have been extensively used in the past to measure the absorbed dose both in phantoms [52] and in animals [53–55]. However, to the best of the authors’ knowledge, this is the first work that compares absorbed doses obtained from ^90^Y–PET quantitative imaging procedures with those obtained from experimental measurements using TL dosimeters. Direct Monte Carlo radiation transport is presently considered to be the most accurate of all currently available dose estimation algorithms [56]. As a consequence, its validation using dosimeters calibrated against absorbed dose primary standards has become even more pressing. Our research has confirmed the accuracy of MC calculations in ^90^Y–PET dosimetry. ${\overline{D}}_w^{meas}$ and ${D}_w^{MCNP}$ are in the ratio 1.088. This difference needs to be further investigated, especially in the light of the well-known uncertainty of MCNP cross sections at extremely low energies [57]. On the other hand, measured doses were in excellent agreement with absorbed doses calculated using a full MC approach, being ${\overline{D}}_w^{\mathrm{meas}}$ and ${D}_w^{\mathrm{Raydose}}$ in the ratio 1.03, well within the stated uncertainties.

The current research acknowledges a few limitations that should be noted to aid interpretation of the result. It is worth noting that the ability of a PET scanner to perform accurate ^90^Y quantification relies on the knowledge of the internal pair production branching ratio. Therefore, precise knowledge of the branch ratio of the 0^+^–0^+^ transition of ^90^Zr is important for an accurate quantification of ^90^Y accumulated inside the target region and detected via PET acquisition. Most recent literature findings report an internal pair production branch ratio as large as (3.186 ± 0.047) × 10^−5^ measured by Selwyn [58] and colleagues using a HPGe detector. This branch ratio value was used in the present study for the absolute calibration of the PET system. It is desirable that in the near future, the current uncertainty on the internal pair production branch ratio (about 1.5%) will be reduced by more accurate experimental measurements [59].

Furthermore, we focussed on absolute quantification and dosimetry on a single cylindrical insert simulating a liver lesion. The extent to which both our methods and results can be generalized to other geometries and different PET scanners certainly requires further investigation. Finally, it is worth noticing that PET calibration measurements should be repeated over a reasonable long period of time to assess the system stability and to evaluate the final uncertainty in the calibration factor. Unfortunately, it was not possible in the present experiment to investigate impact of repeated calibration measurements due to the limited resources available.

Contrary to external beam radiotherapy, in which individual patient dosimetry is mandatory and there are legal requirements for accuracy (within 5% to a reference point), there is a lack of standardization in molecular radiotherapy (MRT), and it suffers from isolated efforts to harmonize quantification and dosimetry approaches. The current main source of uncertainty in internal dosimetry is in taking the step from dose measurements on simple reference geometries to quantitative imaging measurements of the complex and varying geometries of the activity localized in real patients. This passage is essential to comply with EC Directive 2013/59/EURATOM, Article 56, which states that individual dose planning for radiotherapy patients (including MRT) must be enforced in legislation by EU member states by 6 February 2018. It is desirable that in the near future, the final standard uncertainty in absolute quantification in complex phantoms be well below well 5% to ensure the uncertainty in clinical absorbed dose estimations comply with the requirement of 5% to a reference point. Comprehensive guidance has yet to be presented in this field, and there is no doubt that an internationally endorsed protocol on ^90^Y PET/CT quantitative imaging would lead to further advances in this area.

## Conclusions

The results of this research are sufficient to conclude, with some caveats, that patient-specific dosimetry is possible even in a scenario with low true coincidences and high random fraction, as in ^90^Y PET imaging, granted that accurate absolute PET calibration is performed and acquisition times are sufficiently long. In the final analysis, despite Monte Carlo calculations outperforming all dose estimation algorithms, we believe to have gathered ample evidence to recommend the use of the LD algorithm for routine ^90^Y dosimetry based on PET/CT imaging, due to its simplicity of implementation.

## Methods

### Uncertainty in activity measurements

Activity measurements required for the phantom study were performed using the well-type radionuclide calibrator available on site and traceable to the Italian National Institute of Ionizing Radiation Metrology for the geometry being measured (accuracy within ± 5% at *k* = 2 level, as recommended by AAPM report 181). Weightings of liquid solutions were performed with high-precision balances providing accuracies below 0.01%. Therefore, uncertainties associated with volume measurements were considered negligible.

Activity measurements needed for absorbed dose measurements using LiF:Mg,Cu,P TL detectors in a liquid environment were performed using the ENEA-INMRI portable triple-to-double coincidence ratio (TDCR) counter [35], providing a relative standard uncertainty below 1%.

### PET scanner and reconstruction data

^90^Y PET images were acquired with a GE Discovery ST PET/CT scanner (GE Healthcare, Milwaukee, WI, USA) with lower energy threshold at 425 keV. Acquisition time was 16 h for the reference phantom and 30 min per bed for the anthropomorphic phantom. PET images were reconstructed using the standard GE algorithm (full 3D-OSEM VUEPoint HD two iterations, 15 subsets). PET coincidence window is 12 ns, Peak NECR (3D) 38 kcps, energy resolution 20%, and scatter fraction about 35%. PET scanner sensitivity and spatial resolution were assessed in a previous study [16]. PET data were corrected for attenuation, scatter, random, and dead time using manufacturer software. Random and scatter corrections are incorporated into the iterative algorithm [60]. Scatter correction was performed using a fully 3D approach that considers both the axial and trans-axial scatter components, accurately modeling scatter from hot regions in neighboring super slices and outside the scan field-of-view resulting in greater quantitative accuracy (3D model-based scatter correction, MBSC [61]).

### Convolution algorithm

The convolution algorithm allows the absorbed dose following ^90^Y microspheres administration to be calculated by the convolution of the 3D activity concentration matrix *A*(*x*, *y*, *z*)with the 3D dose kernel for ^90^Y, *K*(*x*, *y*, *z*), according to the following equation:

3 $$ {\displaystyle \begin{array}{l}D\left(x,y,z\right)=\frac{1}{\lambda}\left[K\left(x,y,z\right)\otimes A\Big(x,y,z\Big)\right]\\ {}=\frac{1}{\lambda}\iiint K\left(x-{x}^{\hbox{'}},y-{y}^{\hbox{'}},z-{z}^{\hbox{'}}\right)\cdot A\left({x}^{\hbox{'}},{y}^{\hbox{'}},{z}^{\hbox{'}}\right){dx}^{\hbox{'}}{dy}^{\hbox{'}}{dz}^{\hbox{'}}\\ {}\overset{\mathrm{discrete}\kern0.37em \mathrm{space}}{\Rightarrow}\frac{1}{\lambda}\sum \limits_{x\hbox{'}}\sum \limits_{y\hbox{'}}\sum \limits_{z\hbox{'}}\left[K\left(x-{x}^{\hbox{'}},y-{y}^{\hbox{'}},z-{z}^{\hbox{'}}\right)\cdot A\Big({x}^{\hbox{'}},{y}^{\hbox{'}},{z}^{\hbox{'}}\Big)\right]\end{array}} $$

with $\lambda =\frac{\ln (2)}{T_{1/2}}$ decay constant of ^90^Y. Dose calculations were performed using STRATOS, a voxel-based dose engine on the IMALYTICS Research Workstation.

### Local deposition algorithm

Local deposition algorithm has been recently proposed for dose calculations whenever the energy released by charged particles can be assumed to be locally absorbed with in the same voxel as the decay. The other assumption of local deposition algorithm is that the full width at half maximum (FWHM) of the point spread function (PSF) should be larger than the dose point kernel. This is because the output images can be regarded as a convolution of the real object with the PSF of the system. In the ideal case where PSF = Kernel, the imaging system simulates the energy transport among voxels and the output image can be regarded as the absorbed dose map.

The dose at a single voxel, *D*_*voxel*_, can be calculated as the ratio of the energy delivered to the voxel, *E*_*voxel*_, and the mass of the voxel, *m*_*voxel*_. Assuming a voxel density *ρ* = 1 g cm^3^, *m*_*voxel*_[g] = *v*_*voxel*_[cm^3^], with *v*_*voxel*_ voxel volume. In the present study, images were reconstructed using a voxel size as large as 0.273 cm × 0.273 cm × 0.327 cm. Therefore, *m*_*voxel*_ = 0.024371 cm^3^. The energy delivered to the voxel can be calculated as the number of particles totally emitted into the voxel (*N*_*tot*_) times the average energy delivered to the voxel by each particle ($\overline{E}$):

4 $$ {E}_{\mathrm{voxel}}={N}_{tot}\cdot \overline{E} $$

Since microspheres are permanently implanted in the patient with no removal from the region, the effective half-life is simply the radioactive half-life and the number of ^90^Y particles totally emitted into the voxel can be calculated as follows:

5 $$ {N}_{\mathrm{tot}}=\underset{0}{\overset{\infty }{\int }}{A}_0^{voxel}\exp \left(-\lambda t\right)=1.44\cdot {A}_0^{voxel}\cdot {T}_{1/2} $$

With ${A}_0^{voxel}$ initial activity into the voxel at *t* = 0, and *T*_1/2_ half-life of the radionuclide. Regarding the average energy delivered by each particle, $\overline{E}$, the literature value 0.932 MeV can be assumed. Thus, the average energy delivered to the voxel is:

6 $$ {E}_{voxel}=1.44\cdot {A}_0^{voxel}\left[ Bq\right]\cdot {T}_{1/2}\left[s\right]\cdot 0.932\;\left[ MeV\right] $$

${A}_0^{\mathrm{voxel}}$ is obtained from ^90^Y PET images. In fact, as ^90^Y–PET images are calibrated in terms of activity concentration, *C*_voxel_, (i.e., each voxel*—i* is expressed in term of Bq mL^−1^) the activity into the voxel is given by:

7 $$ {A}_{voxel}={C}_{voxel}\left[\frac{Bq}{\mathrm{mL}}\right]\cdot {v}_{voxel}\left[\mathrm{mL}\right] $$

Thus, the dose at a single voxel, *D*_*voxel*_, can be calculated as follows:

8 $$ {D}_{voxel}=\frac{1.44\cdot {C}_{voxel}\left[\frac{Bq}{mL}\right]\cdot {v}_{voxel}\left[ mL\right]\cdot {T}_{1/2}\left[s\right]\cdot \overline{E}\;\left[\mathrm{MeV}\right]\cdot 1.602\cdot {10}^{-13}\left[\frac{J}{\mathrm{MeV}}\right]}{m_{voxel}\left[\mathrm{kg}\right]} $$

9 $$ {D}_{voxel}\left[\mathrm{Gy}\right]={C}_{voxel}\left[\frac{Bq}{mL}\right]\cdot 4.966\cdot {10}^{-5}\left[\mathrm{Gy}\cdot s\cdot \mathrm{mL}\right] $$

### Absorbed dose measurement equation for TLD dosimetry

In the considered experimental geometry the absorbed dose to water was determined from the TLD reading through the following equation:

10 $$ {D}_w= TL\left({E}_{90_Y}\right)\cdot {N}_{D_w}^{60_{Co}}\cdot {k}_{fv}\cdot {H}_{90_Y,{60}_{Co}}^{\exp, \mathrm{ref}}\cdot {k}_{mat} $$

where

- $\mathrm{TL}\left({E}_{{}^{90}Y}\right)$ is the TLD reading after irradiation with ^90^Y beta particles during measurement.
- ${N}_{D_w}^{{}^{60} Co}=\frac{D_w\left({}^{60} Co\right)}{TL\left({}^{60} Co\right)}$ is the TLD calibration coefficient at ^60^Co quality, with *D*_*w*_(^60^ *Co*) absorbed dose to water delivered to the TLD and TL(^60^ *Co*) the TLD reading after irradiation.
- *k*_*fv*_, finite volume effect, is the loss of absorbed dose due to exclusion of radioactivity from the volume occupied by the TLD.
- ${H}_{{}^{90}Y{,}^{60} Co}^{\exp, \mathrm{ref}}=\frac{{\left({D}_w^{90Y}/{D}_{\mathrm{TLD}}^{90Y}\right)}_{\exp \_\mathrm{geom}}}{{\left({D}_w^{60 Co}/{D}_{\mathrm{TLD}}^{60 Co}\right)}_{\mathrm{ref}\_\mathrm{geom}}}$ is the energy and geometry correction factor, given by the ratio of absorbed doses to water and to TLD material at different radiation qualities and different irradiation geometries. This is calculated by the ratios of D_w_/D_TLD_ (TLD to water conversion factor), taking into account differences between measurement conditions (exp_geom) and reference calibration conditions (ref_geom).
- *k*_mat_, correction factor due to the presence of materials other than TLDs, i.e., PMMA stick hosting the three TLDs and the waterproof PMMA envelope surrounding the stick.

All correction factors reported in Eq. 3 were determined using Monte Carlo simulations with MCNP4c code.

## Abbreviations

AVH: Activity volume histograms
dDVH: Differential dose volume histograms
HI: Homogeneity index
LD: Local deposition
MC: Monte Carlo
PET: Positron emission tomography
PMMA: Polymethylmethacrylate
PVE: Partial volume effect
RC: Recovery coefficient
ROI: Region of interest
SIRT: Selective internal radiation therapy
TLD: Thermoluminescent dosimeter
TOF: Time of flight
VOI: Volume of interest
